# Oxidative stability and sensory evaluation of sodium caseinate-based yak butter powder

**DOI:** 10.1038/s41598-022-22629-8

**Published:** 2022-11-21

**Authors:** Anita N. Agyare, Qi Liang, Xuemei Song, Yan Zhang, Jing Yang, Yongqi Shi

**Affiliations:** Functional Dairy Products Engineering Laboratory of Gansu Province, College of Food Science and Engineering, Gansu Agricultural University, Anning District, Lanzhou, 730070 China

**Keywords:** Biochemistry, Biotechnology

## Abstract

Yak butter’s high unsaturated fatty acid level predisposes it to oxidation, hence must be converted into more stable forms like powder. This study aimed to spray dry yak butter using 10% yak butter and four sodium caseinate (NaCas) formulations: sample A: 100% NaCas; sample B: 50% NaCas, 50% lactose; sample C: 75% NaCas, 25% lactose; and sample D: 25% NaCas, 75% maltodextrin. The powders were vacuum and hermetically sealed, and evaluated for oxidative stability, physical and sensory properties during storage at 65 ℃ for 30 days. The results showed that samples B and D had similar and most favorable physical properties (such as, moisture, water activity, particle size, bulk density re-dispersion time, and encapsulation efficiency); though sample B, together with sample C, browned the most during storage. The majority of the sensory panelists preferred samples B and D; observed high caking in samples C and B; and the least whiteness loss and caking in samples D and A but high off-flavors in samples A and C. After storage, peroxide and thiobarbituric acid values of powder samples ranged from 34.98 to 69.54 meqO2/kg and 1.85–9.43 mg MD/kg, respectively, in the decreasing order of A, C, B, and D. Sample D, followed by B, showed the highest radical scavenging activity. Therefore, for optimum yak butter powder physical properties and oxidative stability, 50%:50%, NaCas: lactose, and 25%:75%, NaCas: maltodextrin formulations should be used. This study provides essential knowledge for butter powder processors.

## Introduction

Yak butter powder is a dehydrated butter that serves as a yak butter replacer, hence used to make or flavor baked goods, soups, and snacks. Yak butter contains higher concentrations of unsaturated fatty acids than cow, goat, and sheep butter. For example, it contains 7–21% higher total monounsaturated fatty acids and 3 times the amount of conjugated linoleic acid compared to the other three kinds of butter^1^. This makes it more prone to oxidation and hence important to convert into more stable forms such as powder. Currently, butter powder is one of the dairy industry’s innovations with fast-increasing demand especially from the bakery industry due to its convenience, oxidative stability, longer shelf-life, and emergency preparedness over raw butter. Previous studies on the global butter powder market have estimated market size of about 2.30 billion US dollars in the year 2022 with a robust growth at a compound annual growth rate of 6.1% from 2022 to 2032. These figures solidify the butter powder market’s sustainability and potential growth in coming years^2–4^.

Spray drying (SD) microencapsulation is the coating of active compound droplets to protect them against external deteriorating factors such as oxygen, light, and moisture. Spray dryer atomizes a preformed stable emulsion into fine droplets after which the droplets’ moisture is evaporated using a heated gas stream to produce dried solid particles. The dried powder is then separated from the gas stream and collected^5^. It is worth noting that spray dryer inlet temperatures (from the gas stream) above 180 °C may reduce the quality, polyunsaturated fatty acids, and increase lipid oxidation of the powder produced^6^. While very low inlet temperatures may result in high sticking of fluid feed in the spray dryer inner walls^7^. Spray drying has been extensively used to microencapsulate edible oils due to its many advantages, such as shorter drying times, ability to create a wide range of particle sizes, higher bulk densities, and encapsulation efficiencies, over other techniques such as freeze-drying^2, 8^. Unfortunately, little research has been conducted on dairy butter SD microencapsulation.

The encapsulation material type used in SD is very critical for encapsulation efficiency as it insulates core materials from external degrading factors such as oxygen and moisture, limits volatile losses, blocks premature reactions between core and environment, and enhances their stability^5^. To fulfill these functions, encapsulation material needs to be highly soluble in water, have emulsifying capabilities, and have low viscosity^9^. Sodium caseinate (NaCas) is a milk protein frequently used in microencapsulation. Due to NaCas’s amphiphilic features and high diffusivity, it evenly distributes around the oil surface. These properties results in NaCas’s high solubility, oil emulsifying properties, and quick interfacial coating development^5, 10^. In addition, this study’s authors observed from preliminary research findings that most butter powders on the market are made with non-fat milk solids (primarily comprised of casein or NaCas, probably to give butter powders the dairy flavor^11^ (A. Agyare and Y. Shi, personal communication, 10th November 2021).

In other studies, microcapsules’ quality and functional properties were enhanced when proteins were mixed with carbohydrates as encapsulation material since their demerit features balanced out. For instance, low viscosities of maltodextrin (MD) and lactose (Lac) complement NaCas’ viscosity to achieve higher encapsulation efficiency (EE) when combined. Conversely, other authors have achieved ideal microcapsule features with only one encapsulation material^12, 13^. Previous studies combined NaCas with lactose and maltodextrin and reported high EE, oxidative stability, and favorable microcapsule physical properties. Ixtaina et al.^14^, encapsulated chia oil with NaCas/Lac formula (stored it at 20 ℃ for 180 days) and found that EE and peroxide values were higher than 90% and 50%, respectively, compared to the control. Yang et al.^15^, discovered over 85% EE and small particle sizes, of the order 7.43 μm which increased bulk density and reduced the air space between particles, resulting in reduced oxidation rate and transportation cost, in NaCas/Lac spray-dried *Swida wilsoniana* oil microcapsules. Furthermore, Selim et al.^16^, and Zhu et al.^17^, reported that encapsulation of fish and soya bean oils using MD resulted in 80% and 50% reduction in peroxide value, respectively, compared to their controls. Erbay and Koca^18^, revealed that MD encapsulation of cheese produced powders with enhanced bulk densities, reconstitution properties, and low surface oil content. Also, sensory evaluation panelists in that study found the lowest caking and highest flowability levels in MD cheese powders.

Considering the current popularity of butter powders on the global market, with about 20 major dairy companies involved in its production^3^, limited studies on their characterization have been reported. Therefore, this study aimed at spraying dry yak butter with four NaCas, Lac, and MD formulations to determine the EE and physical technological properties of yak butter powders. Again, this study seeks to determine changes in yak butter powders’ color, oxidative stability, radical scavenging activities, and sensory evaluations during accelerated storage. This will provide important information to dairy processors, butter powder researchers, and developers as it serve as a theoretical basis for the quality control and product development of yak butter.

## Materials and methods

### Emulsion preparation

Fresh yak butter was obtained from Qinghai Golden Qilian Dairy Co. Ltd (Qinghai province China) and encapsulation materials were obtained from Wanwang Biotechnology Co. Ltd (Shanghai, China). Based on preliminary studies and previous research, four microencapsulation formulations were investigated^14, 19^. Yak butter was melted at 50 ℃ and homogenized with different amounts of sodium caseinate (NaCas), lactose (Lac), maltodextrin (MD) with dextrose equivalent of 10, and distilled water at 50 ℃ for 10 min at 10,000 rpm using XHF-D high-speed dispensator, Ningbo Scientz Biotechnology China. Formulations are shown in Table 1.

**Table 1:** Formulations of initial emulsions and spray-dried powders.

Formulations	Initial emulsions					Spray-dried powders					
	Sodium caseinate (%)	Lactose (%)	Malto dextrin (%)	Butter (%)	Total solids (%)	Core: cell wall	Sodium caseinate (%)	Lactose (%)	Malto dextrin (%)	Butter (%)	Total solids (%)
A	20.00			10.00	30.00	1:2	66.67			33.33	100
B (1:)	10.00	10.00		10.00	30.00	1:2	33.33	33.33		33.33	100
C (2:1)	13.40	6.60		10.00	30.00	1:2	44.44	22.22		33.33	100
D (1:2)	6.60		13.40	10.00	30.00	1:2	22.22		44.44	33.33	100

### Spray drying microencapsulation

The MCGS, SD-500/SD-501 from Xiangtan Xiangyi Instrument Ltd (Hunan, China) was used for the spray drying process. The emulsions were fed into a spray dryer at 60 ℃ and dried at 180 ℃ inlet air temperature and 80 ℃ outlet air temperature^9^. The powders were collected, vacuumed and hermetically sealed in polyethylene bags. As previously reported, oxidative stability of oils stored under 65 ℃ for 24 h was equivalent to that of the oils stored at room temperature for a month^20^. Therefore, the powders were stored at 65 ℃ for 30 days, and analyzed for changes in quality parameters after every 10 days.

### Physical properties of powders

#### Yield

Powder yield was calculated as the weight of recovered powder (WP) after the spray drying process compared to the weight of total solids (TS) in feed emulsion.

1 $$Yield \, \% \; = \; \frac{WP}{{TS}} \times 100$$

#### Moisture and water activity

Moisture was determined by oven drying method using AOAC, 2005^21^. Water activity was determined using HD-3A smart water activity meter from Wuxi Huake Instrument Co. Ltd (Jiangsu, China). Following the manufacturer’s instructions, the equipment was calibrated with a mixture of 9 g salt (provided in the kit) and 50 mL water. Yak butter powder (3 g) was analyzed and values were recorded when the difference between two subsequent readings was less than 0.005.

#### Particle size

The volume mean diameter D (4,3) of yak butter powder particles was measured using Malvern’s Mastersizer 3000 laser diffraction particle size analyzer, United Kingdom with dry dispersion module. The butter powder was put into the Aero S dispersion unit while the refractive index of the butter was set at 1.465q. The data from the computer screen was then analyzed to determine the size of the particles that created the scattering pattern.

#### Tapped density

Two (2) g of powder was measured into 10 mL cylinder, tapped on powder’s surface by hand until constant volume was attained and the tapped density (g/cm^−3^) was calculated as weight/volume^9^.

#### Redispersion properties

Yak butter powder (0.5 g) was placed into beaker containing 150 mL distilled water at room temperature (25 ℃) and powder’s complete dispersion time was recorded when powder particles were not floating on top of the water^9^.

#### Encapsulation efficiency (EE)

Powder surface oil was determined by weighing 1 g yak butter powder onto filter paper (No. 4, Whatman, Maidstone, Kent, UK) placed in a pre-weighed flask, and then washed with 5 mL petroleum ether (b.p. 40–60 ℃). The flask was heated at 102 ℃ for 1 h in an oven to evaporate all petroleum ether and the weight of extracted fat was measured as surface oil (SO) in powder. Powder’s total oil (TO) was determined as the ratio of butter weight to powder weight^22^. The EE was calculated by:

2 $$EE\% = \frac{{{\text{TO}} - {\text{SO}}}}{{{\text{TO}}}}$$

#### Color evaluation

Color values of yak butter powder (20 g) placed in Petri dishes were measured using Konica Minolta Chroma meter CR-140 (Tokyo, Japan) to obtain L*a*b* values of each sample. The equipment was calibrated using the manufacturer’s white and blackboards. Using the CIELAB color system, the psychometric index of L* refers to lightness (ranging from black = 0 to white = 100); a* indicated red/green color coordinates, and b* referred to yellow/blue parameters of powder. Color differences, Euclidean distance (ΔE^∗^_ab_), between yak butter powder types in three-dimensional L*a*b* color space was calculated by;

3 $$\Delta {\text{E}}^{*}_{{{\text{ab}}}} = \, \surd \, \left( {\Delta {\text{L}}*} \right)^{2} + \, \left( {\Delta {\text{a}}*} \right)^{2} \, + \, \left( {\Delta {\text{b}}*} \right)$$

where ΔL*, Δa*, and Δb* denote differences between yak butter powder types^23^.

Chroma values were calculated using;

4 $${\text{C }} = \, \surd \, \left( {{\text{a}}*^{{2}} + {\text{ b}}*^{{2}} } \right)$$

where, a* and b* denote color values of the sample^23^.

Browning indices were calculated using;

5 $${\text{BI }} = \frac{{100x\left[ {\frac{{\left( {a + 1.75xL} \right)}}{{\left( {5.645xL + a - 3.012xb} \right)}}} \right]}}{0.17}$$

where, L*, a* and, b* denote sample’s color values^24^.

Yellowing Index was calculated using

6 $${\text{YI }} = 142.86x\frac{b*}{{L*}}$$

where, L* and b* are the sample’s color values^24^.

#### Scanning electron microscopy

Scanning electron microscopy (SEM) by Hitachi Enterprise Co. Ltd., model S-3400N (Dazi City, Japan) was used to study microcapsule morphology. Powder particles were adhered to double-sided adhesive tape, coated with gold (600 Å), and examined in a high vacuum with 5 kV acceleration voltage. Images were captured under 2.00 K magnification^14^.

#### Sensory evaluation

Sensory evaluation was conducted on yak butter powders’ appearance, graininess, aroma, taste, and overall acceptability. Ten trained panelists (5 males and 5 females, aged 20–45) who were familiar with commercial butter powders and well-trained in food sensory assessment evaluated yak butter powders using a quantitative descriptive method on a scale of 1–7 (7 = extremely like, 1 = extremely dislike). Panelists also analyzed stored powders on whiteness, cakiness, flowability, and off-flavor using a scale of 1–7 (1 = none/absent, 7 = highest intensity, and 1 = brown and 7 = white). The panelists analyzed powders without prior information on the various treatments until the end of the evaluations^18^.

#### Extraction of oil

To evaluate oxidative stability of yak butter trapped in microcapsules, hexane was used to extract butter oil from powder. For forty (40) g yak butter powder, 100 mL n-hexane was added and shaken at 250 rpm for 1 h at room temperature. The butter mixture was then shaken for an hour to ensure that all the microcapsules’ fat extraction by the n-hexane and not only the surface oil. The mixture was filtered and n-hexane evaporated using a rotary evaporator at 35 ℃. The extracted fat was stored at – 80 ℃ for further analyses^25^.

### Oxidative stability

#### Peroxide value (PV)

The method by Adjonu et al.^26^, was followed to evaluate the powder’s PV. Two (2) g of extracted fat was dissolved in a 30 mL trichloromethane to glacial acetic acid (2:3) mixture. Then, 1 mL saturated KI solution was added and the mixture was shaken at 80 rpm for 60 s. Subsequently, the mixture was placed in the dark for 5 min and 100 mL of distilled water was immediately added. Titration was carried out with 0.01 mol/L Na_2_S_2_O_3_ until yellow color appeared, and 1 mL (1 g/100 mL) of starch solution was added (color changed to blue) and titration continued until the deep blue color disappeared.

7 $${\text{PV }} = \frac{{{\text{S }} \times {\text{N }} \times { }1000}}{{\text{g}}}$$

where S = ml Na_2_S_2_O_3_ (blank corrected), N = normality Na_2_S_2_O_3_ solution, and g = mass of ghee.

#### Thiobarbituric acid (TBA)

Butter powder TBA value was evaluated using China National Standard, (2017) (CNS), GB_T 35252-2017^27^. The TBA reagent (200 mg) was diluted in 100 mL n-butanol, stored for 12 h at room temperature, and filtered. Then, 200 mg of extracted fat was thoroughly mixed with 25 ml n-butanol. In addition, TBA reagent and 5 ml each of sample solutions were pipetted into centrifuge tubes and heated in a water bath at 95 ℃ for 2 h. Samples were cooled to room temperature and absorbance was measured at 530 nm using a visible spectrophotometer (Shanghai Spectral Instrument Company Limited, model 723, China).

8 $${\text{TBA }}\left( {\text{mgMDA/kg}} \right) \, = \frac{{50\left( {A - B} \right)}}{m}$$

where A = sample absorbance, B = blank absorbance, and m = mass.

### Radical scavenging activities

#### 2,2-diphenyl-1-picrylhydrazyl (DPPH) radical scavenging activity

Extracted fat (0.5 mL) was dissolved in 0.5 mL ethyl acetate and vortexed for 30 s. Subsequently, 0.15 mL fat solution was mixed with 3.350 mL of DPPH solution (0.04 mM in ethyl acetate) in a centrifuge tube. The mixture was vortexed for 10 s, incubated for 30 min in the dark, and absorbance was read at 517 nm using a visible spectrophotometer (Shanghai Spectral Instrument Company Limited, model 723, China). The absorbance of ethyl acetate DPPH solution was used as a reference (control)^28^.

9 $${\text{DPPH}}\% \, = 1 - \frac{{\text{Sample absorbance}}}{{\text{Control absorbance}}} \times { }100{\text{\% }}$$

#### 2,2’-Azinobis (3-ethylbenzothiazoline-6-sulfonic acid) (ABTS) radical scavenging activity

The ABTS radical stock solution was first prepared by reacting equal volumes of 7.00 mM ABTS diammonium salt and 2.45 mM potassium persulfate solution in dark for 13 h. The solution was diluted with 95% ethanol to attain an absorbance of 1.0 nm. Extracted fat (200 µL) solution prepared by dissolving 0.01 g extract in 10 mL 1-butanol was mixed with 6.00 mL ABTS solution; and absorbance at 734 nm was measured after 15 min in the dark using a visible spectrophotometer (Shanghai Spectral Instrument Company Limited, model 723, China). Reference control was determined with 6.00 mL ABTS solution and 200 µL 1-butanol mixture^28^.

10 $${\text{ABTS}}\% \, = 1 - \frac{{\text{Sample absorbance }}}{{\text{Control absorbance}}} \times 100{ }$$

### Statistical analyses

All experiments were repeated in triplicates and analyzed using Excel 2016. Data on sample comparisons were presented as mean ± SD and analyzed using SPSS (Statistical Package for Social Sciences 22.0) software (Chicago, IL, USA) via one‐way analysis of variance (ANOVA), Tukey’s post hoc test, and sample T-tests. *p* < *0.05* between groups was considered statistically significant.

### Experimental approval statement

All experiments were performed in accordance with the relevant guidelines and regulations. All experimental protocols were approved before analyses by Gansu Agricultural University and Gansu Province Functional Dairy Product Engineering Laboratory’s academic review commissions.

### Ethical statement

The sensory evaluation panelists with written informed consent were reviewed and approved by Gansu Agricultural University and Gansu Province Functional Dairy Product Engineering Laboratory’s academic review commissions before analysis.

## Results and discussions

### Physical properties of yak butter powders

#### Powder yield

Yak butter powder yield is very crucial to its economy and commercialization as it defines the efficiency of the spray-drying process. In this study, sample B had the highest yield while sample C had the lowest yield (Table 2). Sample C’s low yield was due to the high sticking of emulsion on spray dryer walls during production (same observation after process repetitions). This phenomenon could imply that the spray dryer’s inlet temperature was low for the effective encapsulation of sample C formulation^5^.

**Table 2:** Physical properties of yak butter powders.

Formulations	A	B	C	D
Powder Yield (%)	62.36 ± 1.33^b^	66.07 ± 0.75^a^	32.69 ± 1.64^d^	60.99 ± 1.16^bc^
Moisture (%)	3.95 ± 0.47^a^	3.01 ± 0.68^c^	3.62 ± 0.10^b^	2.88 ± 0.22^c^
Water activity	0.26 ± 0.01^b^	0.21 ± 0.01^c^	0.34 ± 0.03^a^	0.17 ± 0.02^d^
Particle Size (µm)	9.63 ± 0.09^a^	6.50 ± 0.0^d^	9.03 ± 0.17^b^	7.25 ± 0.14^c^
Tapped density (g/cm^3^)	0.63 ± 0.02^a^	0.52 ± 0.02^d^	0.60 ± 0.01^b^	0.56 ± 0.01^c^
Redispersing time (min)	24.30 ± 0.03^a^	12.05 ± 0.05^c^	20.28 ± 0.08^b^	11.23 ± 0.03^c^
Encapsulation efficiency (%)	82.26 ± 0.83^c^	90.09 ± 1.09^b^	75.40 ± 0.66^d^	93.42 ± 0.58^a^

Data represented as mean ± standard deviation. “a,b,c,d” indicate significant differences within yak butter powders at *p* < *0.05.*

#### Moisture and water activity

Low moisture and water activity (aw) indicate high microbial stability and prevent microcapsules' stickiness and coalescence, thus making them critical indices for microcapsules' shelf-life^29, 30^. In addition, the high moisture content and aw of samples A and C may enhance their hydrolytic rancidity which may produce off-flavors during storage. According to Adawiyah et al.^31^, the hydrolysis rate is higher in foods with moisture content and aw above the monolayer value of 3.55 g water/100 g solid and aw of 0.19. This is due to the lipase enzyme’s ability at this moisture and aw levels to penetrate the lipid monolayer and break down the triglycerides to free fatty acids and glycerols. Furthermore, sample D had the least water moisture and activity (aw), while samples A and C had the highest water moisture and activity (aw) due to maltodextrin (MD)’s lower water-binding nature (non-hygroscopicity) compared to that of NaCas (Table 2). According to Pudziuvelyte et al.^32^, MD-based microcapsules with 30% MD, had lower aw and moisture than those of NaCas, gum Arabic, skim milk, and beta-cyclodextrin powders. The authors reported 2.99–30.00% powder moisture, which is consistent with the results of this study.

#### Particle size

Particle size is very important because it influences microcapsule appearance, unevenness, redispersion, flowability, texture, and overall acceptability^33^. In this study, yak butter powders’ particle sizes ranged from 6.50 to 9.63 µm and sample B had the smallest particle size (Table 2. Yang et al.^15^ reported similar particle sizes (7.43 µm) in their NaCas and lactose encapsulation formulation. Sodium caseinate usually produces smaller microcapsules due to the proteins’ ability to reduce surface tension between the core and encapsulating agent^34^. Contrarily, it was observed in this study that particle size generally increased with an increase in NaCas, especially in samples A and C. This could be attributed to increased emulsion viscosity due to high NaCas concentrations, resulting in larger powder particle diameters^34^. Previous studies showed that MD has lower viscosity (Newtonian fluid of about 25.00 mPa s for 30.00 g/100 mL at 20 °C and 18.30 mPa s zero shear viscosity) than NaCas (25.90 kPa s zero shear viscosity for 29.00 g/100 mL at 20 °C) and NaCas concentrations higher than 40.10 g/100 mL act like solids^22, 35, 36^. Furthermore, MD combinations with proteins (such as casein) have been reported to produce microcapsules with the smallest particle size^34^. Therefore, the high MD level in sample D comparatively decreased emulsion viscosity, which resulted in smaller droplet formations since there was more energy for the emulsification step^22^. Additionally, NaCas has a higher molecular weight (1200–4700 kg/mol) than those of Lac and MD (342 g/mol and 1500 g/mol) respectively^37–39^. Therefore higher NaCas concentrations on samples A and C microcapsules’ surfaces might also contribute to their larger particle sizes.

#### Tapped density

High tapped density is advantageous in the microcapsule industry since more powder can be packed and transported at lower costs compared to low tapped density powders. Tapped density is influenced by factors such as particle size, shape, degree of inter particulate space, and surface properties, with smooth, and even powder particles having higher tapped densities^40^. In this study, yak butter powder tapped densities increased with increasing particle size, with sample A recording the highest followed by C (Table 2) (*p* < *0.05*). This could be attributed to the fact that larger particles have a smaller specific surface area, indicating lesser friction. As a result, the particles flow more easily and the powder compacts more during tapping, leading to higher tapped density. In addition, the high moisture content of samples A and C increased their molecular weight which resulted in higher bulk densities^40, 41^. Therefore, large quantities of samples A and C can be stored in smaller containers which might improve their oxidative stabilities since air trapped in the microcapsules would be significantly reduced^9^. Yak butter powder bulk densities observed in this study were higher than those reported for some previous MD, NaCas, and Lac microcapsules probably due to differences in formulations^9, 22, 41^.

#### Redispersion properties

Microcapsule dispersion time in the water is influenced by particle size and encapsulating agents^9^. From Table 2, redispersion time decreased with increasing particle size mainly due to the higher surface areas of smaller particles. Sample D had the lowest redispersion time, followed by samples B, C, and A. A similar trend was observed by Zhang et al.^9^, who reported 9.23 min and 16.34 min in their smallest and largest microcapsules, respectively. The redispersion time results in this study supported wettability results previously found in MD and NaCas encapsulated catfish oil’s unsaturated fatty acids (8.35–14.60 min) by Hastarini et al.^19^. It was also observed that redispersion time increased with decreasing lactose concentration; probably due to the higher wettability of sugars (Lac) than proteins (NaCas)^18^. Therefore, a higher yak butter powder’s surface lactose concentration results in higher dispersion in water. In addition, the lower redispersion times of samples B and D were due to their higher EE and resultant lower surface oil. Microcapsules with low surface oils exhibit high wettability due to higher inter-particle friction and hydrophobicity which enhances dispersion in water^42^.

#### Encapsulation efficiency (EE)

Higher powder EE is significant for yak butter’s prolonged protection against oxidation during storage^43^. In this study, sample D had the highest EE result and sample C had the lowest EE (Table 2). The yak butter EE results were consistent with the findings of Lu et al.^13^ and Zhang et al.^9^, as the authors reported an EE range of 69.72–94.30% in oils encapsulated with NaCas and MD formulations. However, Böger et al.^22^ reported lower EE (63.47%) in grape seed oil encapsulated with gum arabic and MD formulation. Conversely, Hastarini et al.^19^, recorded 40.91% from catfish oil powder encapsulated with 9:1 NaCas:MD formulation. The lower EE observed in these studies could probably be due differences in encapsulation material formulations as well as larger particles as observed in sample C of this study. During atomization and SD, encapsulation materials entrap smaller oil droplets more efficiently thus producing more stable emulsions with higher breakage resistance. This generates smaller microcapsules with lower surface oil and higher EE^22, 44^. Furthermore, higher EE of samples B and D may be due increased breakage resistance of the microcapsules as a result of the carbohydrates (Lac and MD) compositions on their microcapsules’ surfaces. These carbohydrates increased the hydrophilic character of the microcapsules’ walls which made the butter fat extraction or escape from the microcapsules more difficult, thus enhancing their EE^45^.

#### Color changes

**Figure 1:**
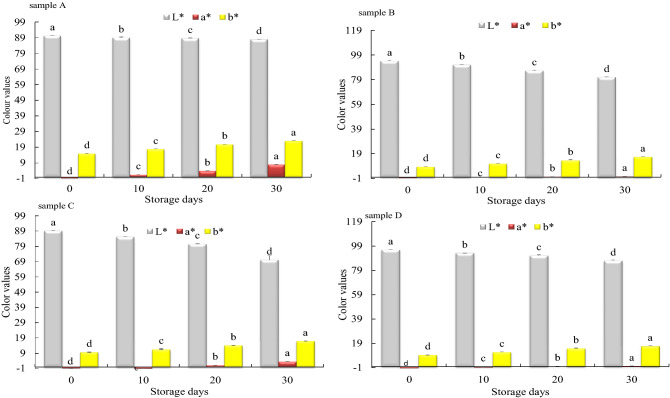
Changes in color values of yak butter powders during accelerated storage. “a,b,c,d” indicate significant differences within yak butter powders at *p* < *0.05.*

**Figure 2:**
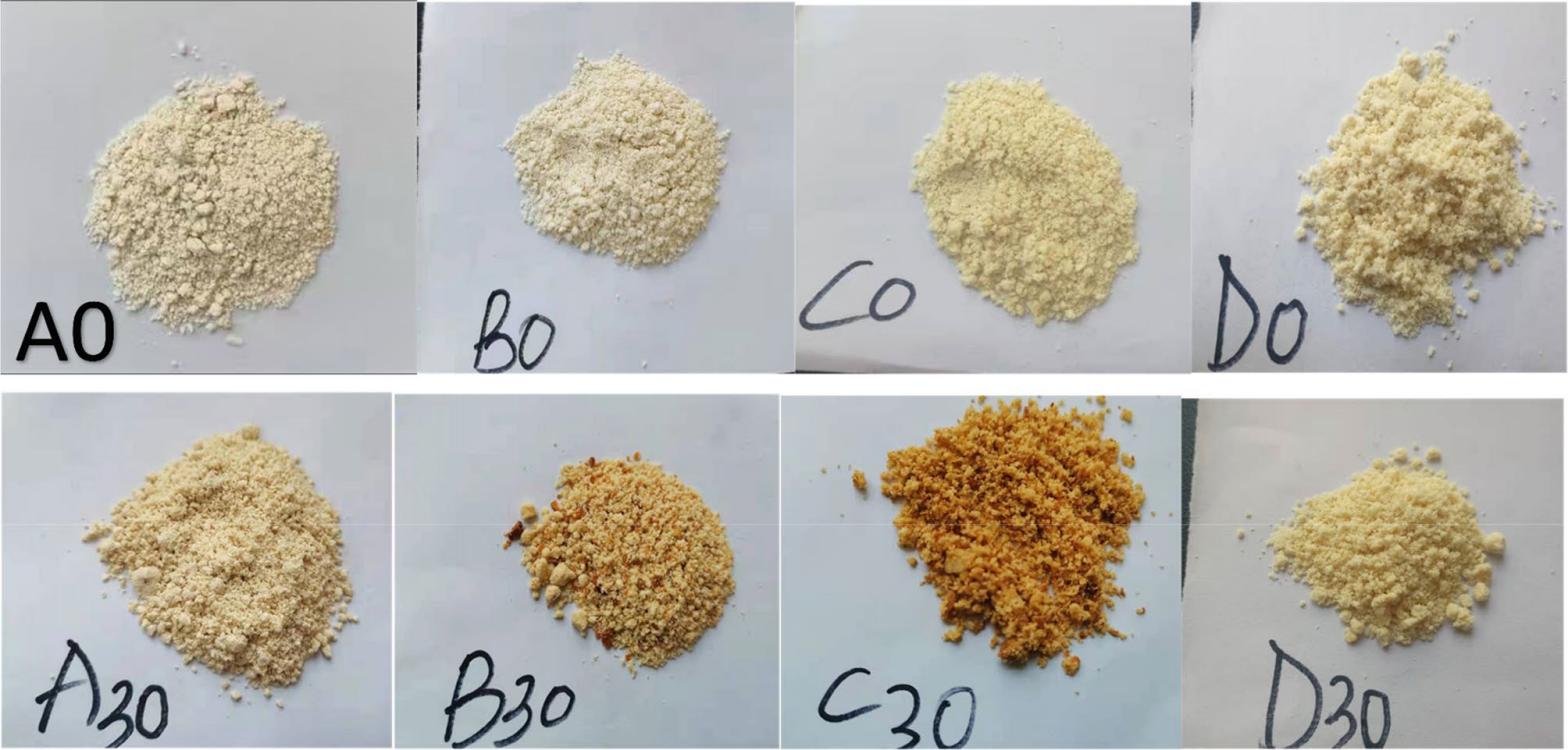
Images of yak butter powders sample A to D at day zero (represented by A0, B0, C0, and D0) and after 30 days of storage (represented by A30, B30, C30, and D30).

Color changes observed were indications of physicochemical reactions that occurred in microcapsules during storage. The results showed that sample A, B, C, and D’s whiteness (L*) parameters decreased (indicating a darker or browner color) from 90.30 to 87.97, 94.38 to 81.16, 89.22 to 70.10, and 95.92 to 86.89, respectively. The a* parameter geared towards color red increased from − 0.69 to 7.97, − 0.64 to 0.66, 0.86 to 3.45, and − 1.49 to 0.42, correspondingly; and b* (yellowness) increased from 14.84 to 23.06, 8.36 to 16.42, 9.70 to 16.96, and 9.53 to 16.74, respectively (Figs. 1 and 2). Thus sample D was whitest; sample C followed by sample B, showed the highest whiteness loss, sample A showed the least whiteness loss, and sample D least browned after storage. The color differences (ΔE^*^_ab_) between samples A and C, as well as samples B and D at day 0, were the only ones that could not be perceived by the human eye since they were below 3^24^ (available in supplementary data).

Yak butter chroma values (colorfulness), brown index (BI) (brown purity), and yellow index (YI) (related to BI) increased significantly in all samples during the storage period (*p* < *0.05*). After storage, sample A’s had the highest chroma value and YI due to its highest a* and L* parameters, respectively (Table 3). Sample D had the least and sample C had the highest BI. Erbay and Koca^18^, previously observed whey and MD cheese powders’ BI increased from 10.67 to 12.50 after 12 storage months at 20 ℃. This study’s higher BI reported in this study was probably due to a difference in encapsulation material as well as higher storage temperature. Similar observation was reported by Nasser et al.^46^, who recounted about 8 BI and 40 BI in micellar casein powders after 11.84 months of storage at 4 ℃ and 60 ℃, respectively.

**Table 3:** Changes in yak butter powders chroma values, brown index, and yellow index during storage.

Indices	Storage days	Samples			
		A	B	C	D
Chroma values	0	14.84 ± 0.26^d^	8.36 ± 0.46^d^	9.70 ± 0.23^d^	9.53 ± 0.20^d^
	10	17.76 ± 0.28^c^	11.01 ± 0.09^c^	11.57 ± 0.15^c^	11.90 ± 0.32^c^
	20	20.78 ± 0.11^b^	13.49 ± 0.14^b^	14.10 ± 0.36^b^	14.77 ± 0.09^b^
	30	23.06 ± 0.38^a^	16.42 ± 0.19^a^	16.96 ± 0.22^a^	16.74 ± 0.56^a^
Brown Index	0	7.26 ± 0.27^d^	11.30 ± 0.24^d^	9.85 ± 0.36	7.75 ± 0.13^d^
	10	9.86 ± 0.09^c^	13.28 ± 0.78^c^	13.11 ± 0.18^c^	9.92 ± 0.52^c^
	20	12.14 ± 0.50^b^	15.45 ± 0.19^b^	20.53 ± 0.41^b^	12.34 ± 0.33^b^
	30	16.25 ± 0.17^a^	20.49 ± 0.25^a^	28.85 ± 0.37^a^	14.01 ± 0.08^a^
Yellow Index	0	23.47 ± 0.37^d^	12.65 ± 0.26^d^	15.54 ± 0.13^d^	14.19 ± 0.44^d^
	10	28.49 ± 0.62^c^	17.25 ± 0.49^c^	19.40 ± 0.73^c^	18.24 ± 0.29^c^
	20	33.48 ± 0.88^b^	22.26 ± 0.16^b^	24.99 ± 0.34^b^	23.16 ± 0.27^b^
	30	37.45 ± 0.26^a^	28.90 ± 0.75^a^	34.57 ± 0.51^a^	27.53 ± 0.80^a^

Data are represented as mean ± standard deviation. “a,b,c,d” indicate significant differences between yak butter powders at *p* < *0.05.*

The yellowing and browning of yak butter powder, especially in sample C, during storage was mainly due to Maillard browning reactions. Maillard browning (MB) reactions occur as a result of chains of non-enzymatic glycation reactions between carbonyl and amino groups in foods^47^. In dairy MB, Lac (carbonyl) reacts with amino acids’ free side chains, especially lysine and methionine. First, an Amadori product is formed, which develops into 3-deoxyosone that progresses through the 4-deoxyosone route to produce melanoidins (browning pigments)^48^. Therefore, yak butter powder’s NaCas and Lac formulations predisposed them to MB during storage and processing. Also, MB levels are influenced by moisture, encapsulation agents’ concentrations, and storage temperature and duration. Compared to sample D, samples B and C’s high NaCas and Lac concentrations resulted in higher BI and YI (MB). Samples A and C’s high moisture most likely increased MB during storage^49^. However, sample C being brownest after 30 days of storage was due to high moisture, and Lac content.

#### Microcapsules morphology

**Figure 3:**
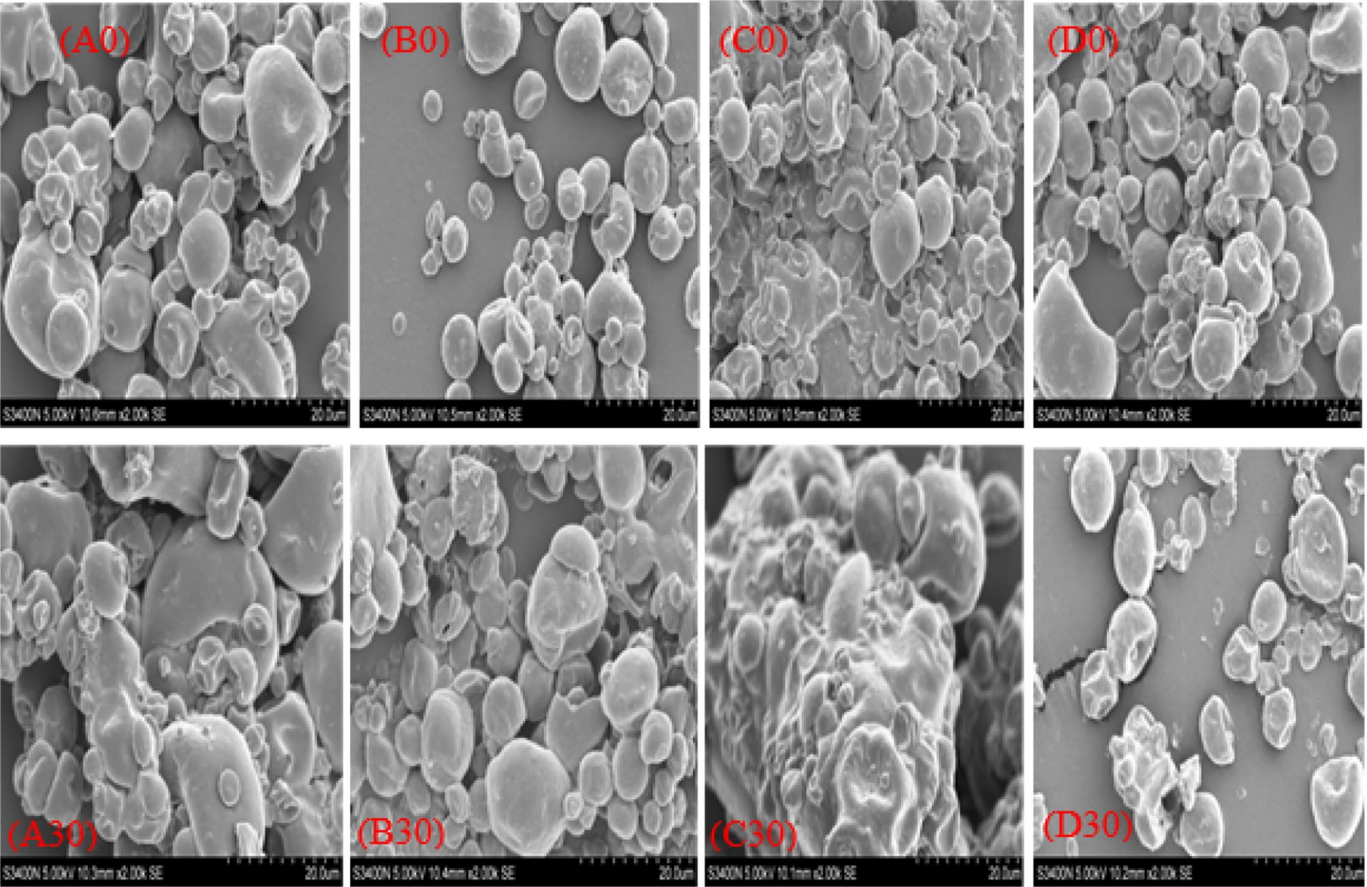
Scanned electron microscope images of yak butter powders sample A to D at day zero (represented by A0, B0, C0, and D0) and after 30 days storage at 65 °C (represented with A30, B30, C0, and D30) using 2000SE magnification.

The Scanning electron microscopy (SEM) results at day zero showed that the majority of yak butter microcapsules were nearly spherical with smooth surfaces (Fig. 3). Sample D microcapsules showed slight shrinkage, while sample C showed slight agglomeration. Most of them had no cracks or holes, indicating complete yak butter encapsulation. After storage, sample C microcapsules completely lost their shapes, showing the highest agglomeration (forming a cake). Samples A, and B’s microcapsules, showed slight agglomerations and the presence of holes, while sample D's microcapsules showed more concavities due to increased surface oils as encapsulation coats lost their integrity^50^. Samples A, B, and C’s increased agglomeration after storage was due to high NaCas hygroscopicity^51^. The speedy water loss and particle surfaces shrinking after SD caused deformity, concavity, and uneven surfaces in butter powder sample^50^. Shrinkage occurs after microcapsule skin hardens, to aid in the spreading of trapped air bubbles inside droplets^52^. Bai et al.^2^, observed similar morphology in kafirin and sodium caseinate soybean oil microcapsules.

#### Sensory evaluation

**Figure 4:**
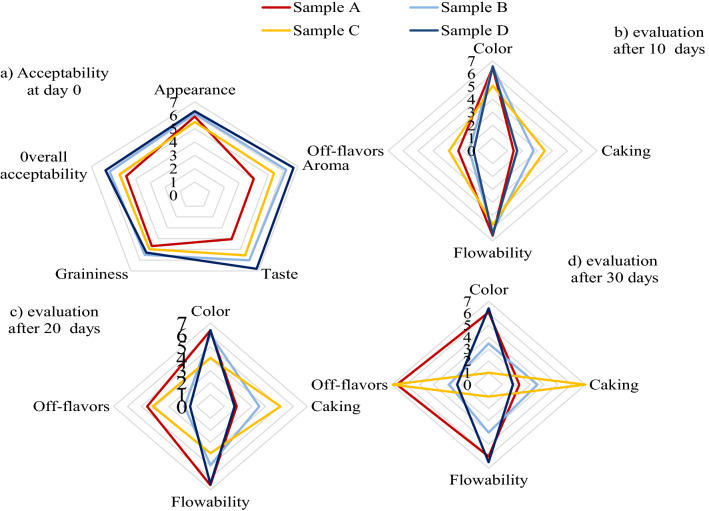
Sensory evaluation of yak butter powders before and during accelerated storage.

**Figure 5:**
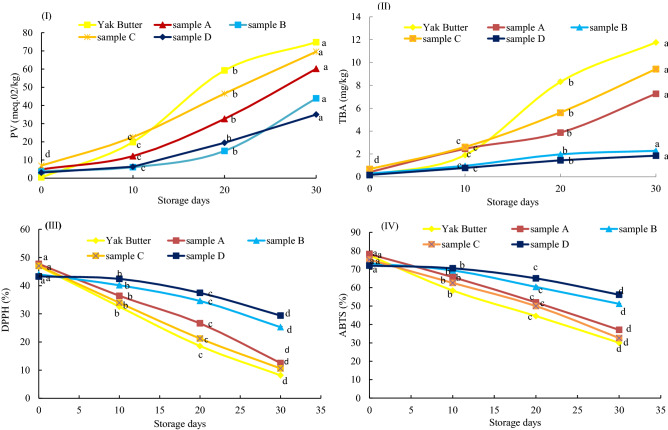
Changes in oxidation stability and radical scavenging activities of yak butter powders during accelerated storage. (1) Peroxide values (PV) (II) Thiobarbituric acid (TBA) test values (III) 2,2-diphenyl-1-picrylhydrazyl (DPPH) radical scavenging activity and (IV) 2,2’-Azinobis(3-ethylbenzothiazoline-6-sulfonic acid) (ABTS) test values. “a, b, c, d” indicate significant differences within yak butter powders at *p* < *0.05.*

In the order of decreasing likeness, panelists moderately liked samples D, B, and A’s appearance but slightly liked that of sample C (Fig. 4), probably due to sample C’s least whiteness (L* value). Panelists liked samples D and B’s aromas moderately, slightly liked sample C’s aroma, and neither liked nor disliked sample A’s aroma. Sample D’s taste was extremely liked, followed by samples B, C, and A, which were slightly disliked, probably due to sugars (Lac and MD) included. Sample B’s graininess was most liked, followed by samples D, C, and A. This could be attributed to particle size differences, as sample A’s largest particle size made it coarsest. Sample D had the highest overall acceptability, followed by samples B, C, and A. Panelists saw no significant changes in samples A and D’s whiteness in the first 20 storage days, while samples B and C’s whiteness declined from “moderately white” and “slightly white” to “slightly brown” and “full brown” respectively (*p* < *0.05*) (Fig. 5). Conversely, panelists ranked samples D and A’s caking at the lowest level during storage (from “absent caking” to “slightly caked”) and only detected a significant difference between them after 30 days of storage (*p* < *0.05*)*.* Sample B was perceived as moderately caked, while sample C was perceived as fully caked.

Caking, which is the formation of aggregates through prolonged undisturbed inter particulate surface contact of microcapsules, is influenced by hygroscopicity, particle size, temperature, moisture, and amorphous content^53, 54^. The amorphous nature of Lac encouraged samples B and C’s powder cake formations under accelerated storage temperature conditions as their glass transition temperatures were exceeded. This increased viscous flow and subsequently strengthened caking in powders. The absence of amorphous sugars in sample A and the non-hygroscopicity of MD in sample D probably suppressed their cake formations^53^. Furthermore, sample C’s high MB rate probably enhanced caking since MB leads to increased sample moisture adsorption, which increased viscous flow and clumping of yak butter powder^55^. It is worth noting that sample C caked completely after 30 days of storage and had to be milled for further analyses, while sample A had soft cakes that were easily broken when pressed.

Yak butter powder flowability negatively correlates with its caking, thus higher caking leads to lower flowability. Therefore, panelists perceived sample C as “absent flowability” and sample D as “moderately flowable,” (ranked highest flowability) after 30 storage days. Panelists only perceive a significant difference in samples A and D’s flowability after 20 storage days (*p* < *0.05*)*.* Panelists also sensed a high decline rate in sample B’s flowability, ranging from “moderately to slightly flowable”. Off-flavor development in powders is an indication of entrapped yak butter rancidity^47^. This study’s panelists only detected off-flavors in sample D after 30 storage days; detected higher off-flavors in sample B after 20 storage days; and sensed the highest off-flavors in samples A and C (almost “full off-flavors”). Panelists’ inability to detect off-flavors in samples D and B was due to their low secondary oxidation products (TBA values). Findings from this study support those of Erbay and Koca^18^, who reported increased caking, decreased whiteness, and flowability of cheese powders during storage. When compared to control and whey formulation, authors observed whiter color, lower caking, and higher flowability in their MD cheese powder.

### Oxidative stability

#### Peroxide value

The results showed that all four yak butter powders initially had higher peroxide (PV) than non-encapsulated yak butter (control) (Fig. 5). However, as storage time progressed, butter powders generally had lower PV than raw butter. The PV values ranged from 4.82 to 60.12 meqO2/kg, 3.85 to 43.84 meqO2/kg, 6.85 to 69.54 meqO2/kg, and 3.03 to 34.98 meqO2/kg, respectively, in samples A, B, C, and D. Sample T-test analyses showed that there was no significant differences between sample B and D’s PVs from 0 to 10 storage days (*p* < *0.05*)*.* In addition, sample D, followed by B, was most effective in inhibiting entrapped butter’s peroxide development. The EE and PV results showed a strong negative correlation (R^2^ = 0.98) implying that high yak butter EE greatly limited peroxide formations as reported in an earlier microencapsulation study^56^. During storage, powder encapsulation materials developed cracks due to surface moisture evaporation. This increased pore size resulted in rapid yak butter outflow onto capsule surfaces (increasing surface oil) and reduced EE. The exposed yak butter’s unsaturated fatty acids reacted with molecular oxygen to form primary oxidation products (PV)^47, 56, 57^. Fats and oils’ oxidative stabilities under 65 ℃ for 24 h is equivalent to one storage month at room temperature^20^. Hence, this study provides an estimation of yak butter powder’s oxidative stability in two and half years. Also, according to Codex standards, permissible butter PV is less than 10 meqO_2_/kg^58^. Thus, samples A and C’s shelf lives were less than 10 months, while those of samples B and D were between 10 and 20 months. Similar PV results were previously been reported on NaCas, Lac, and MD microcapsules during storage^9, 14^.

#### Thiobarbituric acid (TBA) test

With continuous exposure of yak butter powder to molecular oxygen through increased surface oil, peroxides from initial oxidation reactions’ further oxidize to secondary oxidation products. These secondary oxidation products are mainly malondialdehydes (MDA) and other free radicals. They attack and destroy yak butter’s bioactive components and then produce off-flavors such as those detected and unaccepted by the sensory evaluation panelists in this study^47^. The TBA results showed a significant (*p* < *0.05*) increase after every 10 storage days similar pattern as PV results; (Fig. 5)*.* TBA ranged from 0.42 to 7.27 mgMDA/kg; 0.20 to 2.28 mgMDA/kg; 0.58 to 9.43 mgMDA/kg and 0.16 to 1.85 mgMDA/kg in samples A, B, C and D, respectively. It was observed that at day zero, there was not much difference between yak butter and all four powders’ TBA values. Sample T-test analyses showed no significant differences between samples B and D, as well as between samples A and C from 0 to 10 storage days *(p* < *0.05)*. These findings showed that only sample D formulations, followed by sample B formulations, effectively protected yak butter against oxidation throughout the 30 storage days.

According to Spalvins et al.^59^, fats and oils with TBA values less than 1.5 mgMDA/kg are not rancid; those with TBA values ranging from 1.6 to 3.6 mg/kg are slightly rancid but acceptable; while fats and oils with TBA values greater than 3.7 mg/kg are considered rancid and unfit for consumption. Based on this, samples D and B were slightly rancid but acceptable for consumption, and samples C and A were unacceptable for consumption, after 30 storage days. Previous studies by El Ghannam et al.^57^ and Selim et al.^16^, also found increments in fish oil microcapsules’ TBA values during storage. The higher oxidative stability of samples B and D (thus lower PV and TBA values) in this study correlate with their higher radical scavenging activity as subsequently explained.

#### Radical scavenging activity (RSA)

Radical scavenging activity is the elimination of unstable free radicals produced during chains of oxidation reactions in foods. This function is very critical to yak butter powder safety and shelf life as oxidation leads to sensory quality and nutrient losses, and adverse health effects such as diarrhea and cancer^47^. In this study, DPPH values significantly (*p* < *0.05*) decreased in all samples with increasing storage time (Fig. 5)*.* Samples A, B, C, and D’s DPPH values decreased from 47.75 to 12.54%; 44.01 to 25.26%; 47.03 to 10.62%; and 43.27 to 29.38%, respectively. There was no significant difference between control, and samples A and C’s DPPH values, as well as between samples B and D's DPPH values from 0 to 10 storage days (*p* > *0.05*). A similar trend was observed in ABTS results (Fig. 5). The ABTS of samples A, B, C, and D decreased from 78.25 to 37.06%; 73.25 to 51.19%; 75.18 to 32.61%; and 71.98 to 56.16%, respectively. However, from 10 to 30 days, sample T-test analysis revealed a significant difference between samples B and D.

The RSA involves a series of oxidation and reduction reactions where yak butter powder antioxidants stabilize free radicals by donating hydrogen atoms or electrons, thus converting them to non-reactive species. The high RSA of samples A and C at day zero could be due to higher peptide levels owing to higher NaCas concentrations compared to levels in samples B and D. NaCas peptides were able to donate more electrons to free radicals, thus terminating radical chain reactions^60–62^. Also, lower RSA in sample D at day zero was probably due to the dilution effect of MD^60^. However, as storage time increased, sample C, followed by A’s RSA, declined at a faster rate than samples B and D’s RSA. This could be attributed to surpassing production rates of peroxides, MDA, and other reactive species through oxidation. Hence, samples A and C antioxidants were spent at faster rates on scavenging these accumulated radicals^47^. This study supports findings by González-Peña et al.^63^, who observed about 60% RSA loss after MD’s storage and gum arabic encapsulated carotenoids.

## Conclusion

This study revealed that, depending on formulation composition, NaCas-based formulations produced yak butter powders with ideal quality properties. Generally, samples B (50% NaCas, 50% lactose) and D (25% NaCas, 75% maltodextrin) showed the most favorable physical properties with no appreciable difference between their results, followed by sample C (75% NaCas, 25% lactose), and then sample A (100% NaCas), which exhibited the poorest physical quality. Browning and yellowing increased in all yak butter powder samples during accelerated storage, with NaCas and Lac formulations showing the highest levels. In terms of sensory evaluation, most panelists preferred samples D and B’s organoleptic properties at day zero. According to panelists, during storage, sample D showed the highest stability against browning, caking, and development of off-flavors. Sample D, followed by B, A, and then C, showed the highest oxidative stability and RSA during storage. In a nutshell, although NaCas conferred some beneficial characteristics on yak butter powder, concentrations higher than 50% (of encapsulation material) were detrimental to the overall quality, acceptability, and oxidative stability of yak butter powder. For optimum physical and sensory properties and oxidative stability of yak butter powder, it is recommended to use samples B (50% NaCas, 50% lactose) and D (25% NaCas, 75% maltodextrin) formulations.

## Supplementary Information

Supplementary Figure S1.

## Data Availability

Data can be made available upon reasonable request from the corresponding author at liangqi@gsau.edu.cn**.**
