# Supplementary material for: Oxidative stability and sensory evaluation of sodium caseinate-based yak butter powder
Source: Sci Rep. 2022 Nov 21;12:20062. doi: 10.1038/s41598-022-22629-8 (PMC9681766; doi:10.1038/s41598-022-22629-8)
Supplement: Supplementary file 1 — Supplementary Figure S1. [file 41598_2022_22629_MOESM1_ESM.docx]

Fig 3. Color differences between yak butter samples. Bars A, B, C, D represent samples A, B, C, and D. “a,b,c,d” indicate significant differences within yak butter powders at *p < 0.05*
